# Unveiling the molecular features, relevant immune and clinical characteristics of *SIGLEC15* in thyroid cancer

**DOI:** 10.3389/fimmu.2022.975787

**Published:** 2022-09-09

**Authors:** Xiaofeng Hou, Chao Chen, Xiabin Lan, Xiaodong He

**Affiliations:** ^1^ The Second Clinical Medical College, Lanzhou University, Lanzhou, China; ^2^ Department of Head & Neck Oncology Surgery, the Cancer Hospital of the University of Chinese Academy of Sciences (Zhejiang Cancer Hospital), Hangzhou, China; ^3^ Key Laboratory of Head & Neck Cancer Translational Research of Zhejiang Province, Hangzhou, China

**Keywords:** thyroid cancer, *SIGLEC15*, mRNA expression, clinical characteristics, immune dysfunction

## Abstract

The groundbreaking research work about *SIGLEC15* has raised it as a potential promising target in cancer immunotherapy. Unfortunately, the role of *SIGLEC15* in thyroid carcinoma (THCA) remains obscure. Public and home multi-omics data were collected to investigate the role of *SIGLEC15* in THCA in our study. *SIGLEC15* was upregulated in THCA tumor tissue compared to nontumor tissue in both mRNA and protein levels; gene set enrichment analysis (GSEA) results showed that high *SIGLEC15* mRNA expression was positively correlated to many immune pathways. Results of the examination of immunological landscape characteristics displayed high *SIGLEC15* mRNA expression that mainly positively correlated with a large number of cancer immunity immunomodulators and pathways. In addition, upregulation of *SIGLEC15* was positively correlated with an enhanced immune score, stromal score, and estimate score. However, higher *SIGLEC15* mRNA also met high immune exhausted status. The majority of CpG methylation sites negatively correlated with *SIGLEC15* mRNA expression. Analysis of clinical characteristics supported increased *SIGLEC15* expression that was positively correlated with more extrathyroid extension and lymph node metastasis. We observed different single nucleotide variant (SNV) and copy number variation (CNV) patterns in high and low *SIGLEC15* mRNA expression subgroups; some vital DNA damage repair deficiency scores addressed a negative correlation with *SIGLEC15* mRNA expression. We also found that some commonly used chemotherapy drugs might be suitable for different *SIGLEC15* mRNA expression subgroups. This study highlighted the vital role of *SIGLEC15* in THCA. Targeting *SIGLEC15* may offer a potential novel therapeutic opportunity for THCA patients. However, the detailed exact cellular mechanisms of *SIGLEC15* in THCA still needed to be elucidated by further studies.

## Introduction

Thyroid cancer is a common endocrine system tumor, and its incidence rate has been increasing steadily in recent years (1–3). What was worse is that it was reported that thyroid cancer is the fifth most common cancer in women (4). Although the increase in incidence rate could be largely attributed to the improvement in the detection and diagnosis technology of papillary thyroid cancer (PTC) (diameter <2 cm), the ratio of more invasive PTC (diameter 2.1–4.0 cm and >4 cm) increased by 1.5–5 times in the past 30 years (5), even if the majority of them had excellent long-term prognoses. However, some kind of thyroid cancer may also exhibit very aggressive behavior, and the mortality rate remains stubbornly high (6). There are still a small number of patients with advanced differentiated, poorly differentiated, and undifferentiated thyroid cancer with high mortality. To date, the most efficacious approach is targeted therapy with or without surgical resection, but the curative effect is still poor. Therefore, there is an urgent need for newer therapies.

More and more studies believed that the tumor microenvironment (TME) was an essential factor that affected tumor formation, development, and prognosis (7–10). Therefore, a lot of effort has been stimulated to identify immune factors that contribute to the prognosis of cancer patients. Checkpoint inhibitors, chemotherapy drugs, radiotherapy, and antiangiogenic drugs all enhanced T-cell infiltration in the tumor microenvironment (11). Immunotherapy has become the fourth primary treatment of tumors following surgery, radiotherapy, and chemotherapy. Immune checkpoint inhibitors, especially, have accomplished unprecedented success in the clinical treatment of multiple cancer types. There was also a growing body of cancer clinical trials that were approved by the US Food and Drug Administration (FDA) in which a single-agent checkpoint inhibitor or a kind of combination of checkpoint blockades was a treatment (12–14), demonstrating the breadth of interest from scientists and pharmaceutical factories in immuno-oncology and the great potential for additional immunotherapeutics.

Immune checkpoint-blocking therapy such as cytotoxic T lymphocyte-associated antigen 4 (*CTLA4*) (15, 16), programmed cell death protein 1 ligand 1 *(PD-L1)*, and programmed cell death 1 *(PD-1)* have been widely used in many types of solid tumors (17, 18). Although anti-*PD-1*/*PD-L1* therapy was the most famous and clinically effective immunotherapy, its effectiveness in human solid tumors remains only 20%–30% (19). In recent years, immunotherapy has been successfully applied to the treatment of advanced differentiated thyroid cancer and anaplastic thyroid cancer and changed the treatment paradigm (20, 21).

*SIGLEC15*, an alias of *CD33L3* and *HsT1361*, belonged to the sialic acid-binding immunoglobulin-like lectin family. Angata et al. (22) first identified *SIGLEC15* in 2007 and inferred that it probably played a conserved regulatory role in the immune system of vertebrates. The following studies demonstrated that *SIGLEC15* played an important role in the development and differentiation of osteoclastogenesis, and it could also act as a potential therapeutic target with its versatile role of suppressed bone resorption but also facilitated bone remodeling (23–25). Jaeger et al. (26) identified *SIGLEC15* as a susceptibility factor in recurrent vulvovaginal candidiasis. Wang et al. (27) first supported that *SIGLEC15* could be an immune suppressor and potential target for normalization cancer immunotherapy by using a genome-scale T-cell activity array in 2019, and they also revealed upregulation of *SIGLEC15* on various cancer types. Moreover, *SIGLEC15* had unique molecular features when compared with the majority of known checkpoint inhibitory ligands and a mutually exclusive expression with *PDL1*, proposing that it could be a critical immune evasion mechanism in PD-L1-negative patients (27). *SIGLEC15* was proven to be an immune suppressor in the premetastatic lymph node of colorectal cancer (28). Several studies displayed the complicated function of *SIGLEC15* and validated that *SIGLEC15* could act as a potential immunotherapeutic target for pancreatic ductal adenocarcinoma (29, 30). *SIGLEC15* shaped a non-inflamed TME and predicted the molecular subtypes in bladder cancer (31). Furthermore, *SIGLEC15* acted as a mediator of *LINC00973*t to suppress immune in clear-cell renal cell carcinoma (32). Most importantly, in a phase I clinical trial of *SIGLEC15*-positive patients who were diagnosed with advanced/metastatic solid tumors refractory or resistant to currently available therapies with a tumor proportion score Tumor Proportion Score (TPS) PDL1 score <50% could benefit from NC318 (anti-*SIGLEC15* antibody) (33).

In the present study, we aimed to decipher the comprehensive picture of the role of *SIGLEC15* in thyroid carcinoma (THCA) by datamining the well-known multi-omics databases, such as The Cancer Genome Atlas (TCGA) and Gene Expression Omnibus (GEO), and validated it in our own dataset by experiments.

## Materials and methods

### Data acquisition

We obtained TCGA and Genotype-Tissue Expression (GTEX) RNA sequencing data (FPKM), clinical data, and DNA methylation data from xenabrowser (https://xenabrowser.net/datapages/) (34, 35), and FPKM values were transformed to log2(TPM + 1) values. *SIGLEC15* mRNA differential expression from the Pan-Cancer Analysis of Whole Genomes (PCAWG) was completed by R package UCSCXenaShiny (36). GEO datasets (GSE3467, GSE3678, GSE29265, GSE33630, GSE60542, GSE65144, GSE97001) were downloaded from GEO database (https://www.ncbi.nlm.nih.gov/geo/) (37). R package limma was used to get the mean value of repeated probes in GEO datasets (38). Wilcoxon rank-sum test was used to compare the differential expression of *SIGLEC15* between tumor and normal samples in both The Cancer Genome Atlas, and GEO is Gene Expression Omnibus and GEO datasets; R package ggpubr and ggplot2 were used to visualize the difference (38). R package survival was used to explore the relationship between *SIGLEC15* expression and overall survival. Somatic mutation MAF (Varscan2 version) file was downloaded from the GDC data portal (https://portal.gdc.cancer.gov/) and presented gene mutation between high and low *SIGLEC15* (cut by median value of *SIGLEC15*) (39). Copy number information was harvested from Masked Copy Number Segment by R package TCGAbiolinks (40), then separated into two files by high and low *SIGLEC15* (cut by median value of *SIGLEC15*); these two files were used as input for the online tools GenePattern module GISTIC_2.0 (version 6.15.28) (https://www.genepattern.org/#) to visualize the copy number variation (CNV) difference (41). We downloaded the genomic and molecular landscape of DNA damage repair deficiency scores file (42) and explored the correlation with *SIGLEC15* mRNA expression. Online database Tumor Immune Single-cell Hub (TISCH) was conducted to explore the expression cell type of *SIGLEC15* (43).

### *SIGLEC15* mRNA expression correlation with DNA methylation and N6-Methyladenosine regulator mRNA expression

We extracted DNA methylation site beta values 2 kb upstream to 0.5 kb downstream of the transcription start site (TSS) of gene *SIGLEC15*, then conducted a Spearman correlation analysis between *SIGLEC15* DNA methylation and mRNA expression (including gene level and site level; gene level is the mean value of all site values). We also explored the correlation between *SIGLEC15* mRNA expression and N6-methyladenosine (m6A) genes (including 13 readers, eight writers, and two erasers) (44).

### Functional enrichment analysis

We used Wilcoxon rank-sum test to find the differentially expressed genes between high and low *SIGLEC15* (cut by median value of *SIGLEC15*) groups and visualized by R package pheatmap. Gene set enrichment analysis (GSEA) software (GSEA v4.2.3) (45, 46), h.all.v7.5.1.symbols.gmt, and c5.go.v7.5.1.symbols.gmt were harvested from msigdb (http://www.gsea-msigdb.org/gsea/downloads.jsp) and then for GSEA with the high and low *SIGLEC15* mRNA expression groups. Significant signaling pathways were selected by criteria false discovery rate (FDA) <0.25 and p-value <0.05.

### Protein–protein interaction (PPI) network and hub genes

Significant differentially expressed genes between high and low *SIGLEC15* (cut by median value of *SIGLEC15*) groups were based on the criteria of false discovery rate <0.05 and absolute value log2 fold change >1, then the selected genes were input into SRTING (v11.5, http://string-db.org/) for the retrieval of protein–protein interaction network information (47). A cutoff of 0.4 for minimum interaction score was set to get the biological functions with disconnected nodes hidden from the network, and the interaction file acted as input for Cytoscape3.9.1 to visualize the interaction network of these proteins (48); plug-in CytoHubba was applied to get hub genes with default parameters (49).

### Evaluation of the immunological landscape characteristics with *SIGLEC15* of the thyroid carcinoma

To decipher the immunological landscape characteristics of the TME in THCA, we firstly gained 122 immunomodulator genes [major histocompatibility complex (MHC), receptors, chemokines, and immune stimulators] (50), 47 immune checkpoint (ICP) genes, and 25 immunogenic cell death (ICD) genes (51). We displayed the different expressions between high and low *SIGLEC15* (cut by median value of *SIGLEC15*) groups or correlation with gene *SIGLEC15*. The activity of the cancer immunity cycle data was obtained from online website TIP (http://biocc.hrbmu.edu.cn/TIP/index.jsp) (52) and immune features from the online website iAtlas Explorer (https://isb-cgc.shinyapps.io/iatlas/) were downloaded (53) and then were visualized by R package ggpurb between high and low *SIGLEC15* (cut by median value of *SIGLEC15*) groups. R package estimate was used to output the estimated levels of infiltrating stromal and immune cells and calculated stromal score, immune score, and estimate score (54). The TIMER website (https://cistrome.shinyapps.io/timer/) was utilized to validate the influence of *SIGLEC15* expression on immune cell infiltration in THCA (55). We also used single-sample gene set enrichment analysis (ssGSEA) to compare the immune infiltration scores between high and low *SIGLEC15* (cut by median value of *SIGLEC15*) groups (56). The Tumor Immune Dysfunction and Exclusion (TIDE) score and exclusion score were evaluated using an online database (http://tide.dfci.harvard.edu/) (57).

### Tissue microarray analysis

This study was approved by the institutional review board of Zhejiang Cancer Hospital. The informed consents were signed from all subjects in the study. The tissue microarray (TMA) chips were obtained from Wuhan Xavier Biotechnology Co., Ltd. A total of 110 thyroid cancer tissue specimens and 54 adjacent tissue specimens were obtained; each formalin-fixed paraffin-embedded block was cut into 4-µm-thick sections for arraying. *SIGLEC15* antibody (GTX32061, GeneTex, CA, USA) was used for immunohistochemistry (IHC); representative cancer tissue areas were marked on hematoxylin–eosin-stained slides, and TMA construction sampling was performed using tissue chip scanner (3DHistech^®^, Pannoramic MIDI, Hungary). The Densito Quant software in Quant Center was used to automatically identify and set all dark brown on the tissue section as strong positive, brown yellow as medium positive, light yellow as weak positive, and blue nucleus as negative. Then, each tissue point was identified; the strong positive, medium positive, weak positive, and negative areas (unit: pixel) were analyzed; and the positivity percentage and histochemistry score (H-score) were calculated.

### Evaluation of potential chemotherapy drugs to *SIGLEC15* mRNA expression

Cellminer™ database [Version: 2022.1 (database: 2.8.1)] was used for the research of pharmacological data of the NCI-60 cancerous cell lines (58).

### Statistical analysis

Statistical analysis was finished with R software (v4.1.1, https://www.r-project.org/) and its corresponding packages. Comparison between two groups was conducted utilizing Wilcoxon rank-sum test, and Kruskal–Wallis test was carried out for normal multiple groups. Spearman correlation test was adopted to determine the correlation between variables. Fisher exact test was performed to analyze the correlation between *SIGLEC15* and clinicopathologic characteristics. p-value <0.05 was set as the threshold; if not specially noted, ns, *, **, ***, and **** stand for p-value >0.05, p-value <=0.05, pvalue <=0.01, pvalue <=0.001 and pvalue <=0.0001, respectively.

## Results

### Analysis of expression of *SIGLEC15* in thyroid carcinoma samples

**Figure 1:**
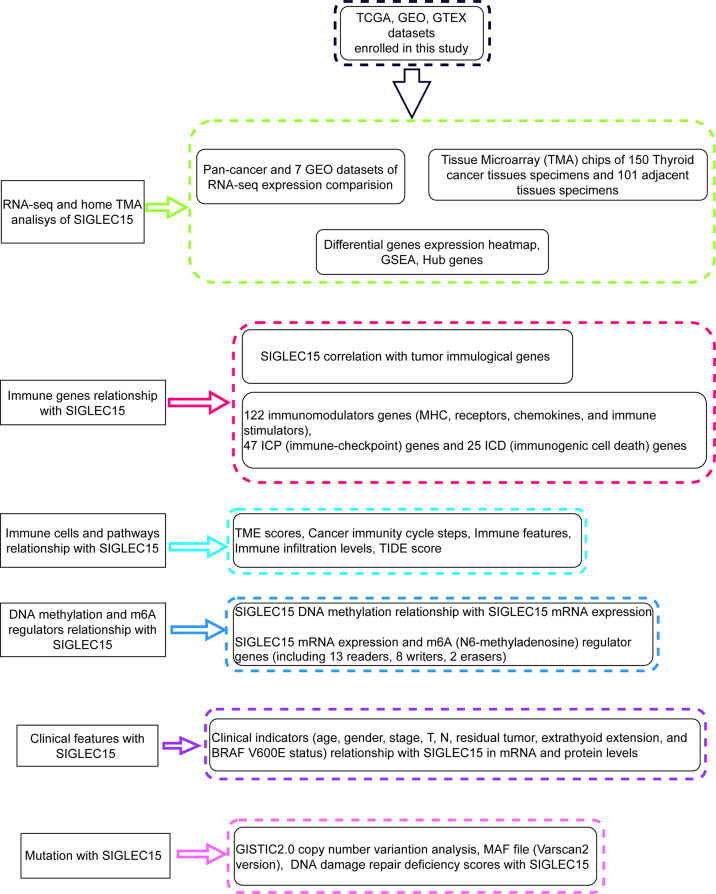
The flow diagram of the study.

**Figure 2:**
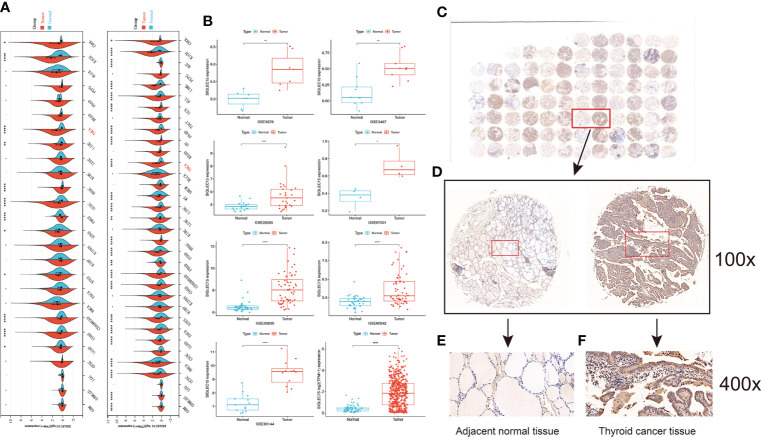
Analysis of the expression of *SIGLEC15*. **(A)** Pan-cancer mRNA expression of *SIGLEC15* between tumor and normal tissues from The Cancer Genome Atlas, and GEO is Gene Expression Omnibus and Genotype-Tissue Expression database. **(B)** mRNA expression of *SIGLEC15* between tumor and normal tissues from GEO and The Cancer Genome Atlas, and GEO is Gene Expression Omnibus database. **(C)** A classic pair of samples. **(D–F)** Left sample was adjacent normal tissue. Right sample was thyroid carcinoma tissue. Thyroid carcinoma tissue has higher *SIGLEC15*-positive staining than the adjacent normal tissue. ns, *, **, ***, and **** stand for p-value >0.05, p-value <=0.05, p-value <=0.01, pvalue <=0.001 and pvalue <=0.0001, respectively.

**Figure 1** showed the workflow of this study, which was presented for *SIGLEC15* differential expression, immune genes, cells, pathways, immune infiltration scores, clinical features, mutations, CNV, DNA methylation, and m6A genes with *SIGLEC15*. After a comprehensive analysis of the expression data from The Cancer Genome Atlas, and GEO is Gene Expression Omnibus, Genotype-Tissue Expression, and PCAWG database, we found that *SIGLEC15* was highly expressed in THCA compared with normal tissues in all databases (**Figure 2A**; **Supplementary Figure S1A**). In addition, *SIGLEC15* mRNA expression from database The Cancer Genome Atlas, and GEO is Gene Expression Omnibus of THCA in tumor and normal samples was shown in **Figure 2B**. Paired tumor and normal samples in **Supplementary Figure S1B**, *SIGLEC15* was also found to be highly expressed in tumor samples in gene expression profiles (GSE3467, GSE3678, GSE29265, GSE33630, GSE60542, GSE65144, GSE97001) from the GEO database in **Figure 2B**. The expression level and the positive rates of *SIGLEC15* were compared between thyroid cancer tissue and adjacent normal tissue samples by immunohistochemistry on microarray. **Figures 2C, D** were a representative area of TMA, a classic pair of samples. Adjacent tissue had lower positive staining (**Figure 2E**) than thyroid cancer tissue (**Figure 2F**); the concrete H-score was in **Table 1**.

**Table 1:** *SIGLEC15* expression levels in different pathological tissues.

	N	H-Score ( $\bar{X}\pm \text{S}$ )	t-value	p-value
Tumor tissue	110	95.18 ± 29.10	6.124	0.001
Paratumor tissue	54	64.92 ± 31.0		

H-Score, histochemistry score.

### Differential genes, signaling pathways, and hub genes associated with *SIGLEC15* mRNA expression groups

**Figure 3:**
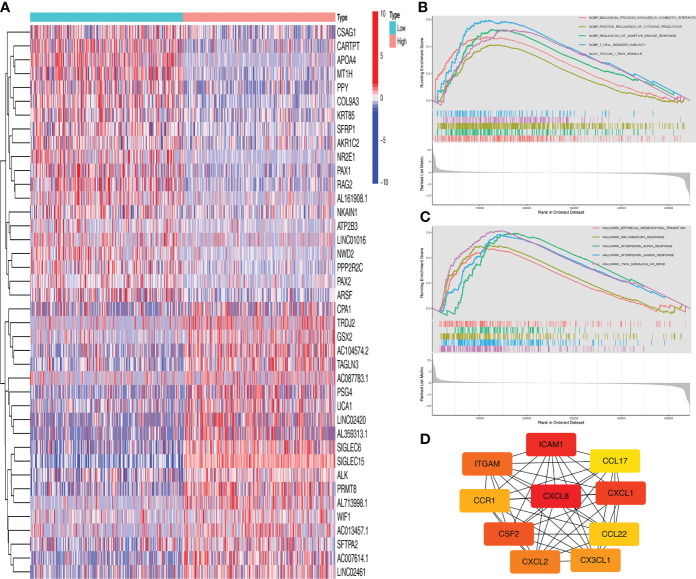
Functional enrichment analysis of *SIGLEC15*. **(A)** Top 20 differentially expressed genes between high and low *SIGLEC15* groups. **(B, C)** GSEA for the signaling pathways activated in the high *SIGLEC15* mRNA expression group with **(B)** GO pathways and **(C)** HALLMARK pathways. **(D)** Hub genes related to *SIGLEC15*.

We obtained significant differentially expressed genes between high and low *SIGLEC15* groups. The 20 most highly and lowly expressed genes were presented, and we noted that the SIGLEC family genes *SIGLEC15* and *SIGLEC6* were in the 20 most highly expressed genes in tumor samples (**Figure 3A**). We further analyzed the signaling pathways involving *SIGLEC15 via* GSEA; high *SIGLEC15* mRNA expression was positively correlated with pathways such as the regulation of adaptive immune response, positive regulation of cytokine production, T cell-mediated immunity (**Figure 3B**), inflammatory response, interferon alpha response, and interferon gamma response (**Figure 3C**). Thereafter, we used the methods mentioned above to identify hub genes; 10 hub genes were harvested, as **Figure 3D** presented; several CXC family genes were in the hub gene list, such as genes *CXCL1*, *CXCL2*, and *CXCL8* (**Figure 3D**).

### Immunological and biological significance of *SIGLEC15* in thyroid carcinoma

**Figure 4:**
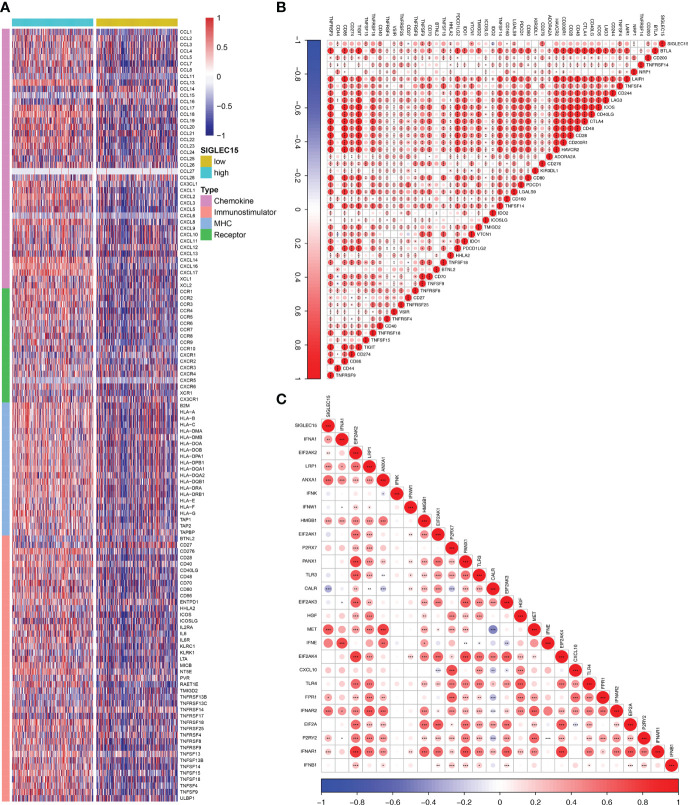
Immunological gene patterns related to *SIGLEC15*. **(A)** Differential expression of 122 immunomodulators (chemokines, receptors, MHC, and immunostimulators) between the high and low *SIGLEC15* groups. **(B)** ICP modulator relationship with *SIGLEC15*. **(C)** ICD modulator relationship with *SIGLEC15*. ns, *, **, and *** stand for p-value ≤0.05, p-value ≤0.01, pvalue ≤0.001.

**Figure 5:**
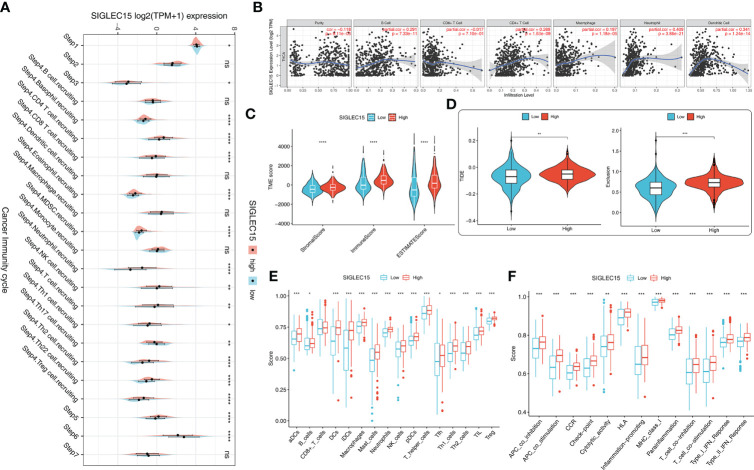
Immunological cell patterns related to *SIGLEC15*. **(A)** Differences in the seven steps of the cancer immunity cycle between the high and low *SIGLEC15* groups. **(B)** *SIGLEC15* was associated with immune cell infiltration in THCA obtained from the TIMER database. **(C)** TME scores were compared between the high and low *SIGLEC15* groups. **(D)** Scores were compared between the low and high *SIGLEC15* groups in TIDE score and Exclusion score. **(E)** Enrichment scores for 16 immunocytes were compared between the low and high *SIGLEC15* groups. **(F)** Enrichment scores for 13 immune-related pathways were compared between the low and high *SIGLEC15* groups. ns, *, **, ***, and **** stand for p-value >0.05, p-value <=0.05, p-value <=0.01, pvalue <=0.001 and pvalue <=0.0001, respectively.

The majority of 122 highly expressed immunomodulators were found in the high *SIGLEC15* mRNA expression group, such as MHC family member genes, CXC family member genes, CXC chemokine receptors that played important roles in cancer immunity (**Figure 4A**), and ICPs and ICD genes, which played critical roles in modulating the host antitumor immunity. *SIGLEC15* had a positive correlation with most of the ICPs, and some had a significant positive correlation (e.g., *CD200*, *CD276*, *CD40*) but had no significant correlation with *PDCD1 (PD-1)* (**Figure 4B**). *SIGLEC15* also had a significant positive correlation with some ICDs (e.g., *ANXA1*, *MET*) and significant negative correlation with *CALR* (**Figure 4C**). We also found a significant positive correlation between *SIGLEC15* and *CD44* (**Supplementary Figure S1C**; **Supplementary Table S1**). Systematically tracking the activity of anticancer immunity and the extent of tumor-infiltrating immune cells were important for cancer immunotherapy. The majority of the steps of the cancer immunity cycle were found to be significantly upregulated, including step 3 (priming and activation), step 5 (infiltration of immune cells into tumors), and most parts of step 4 (trafficking of immune cells to tumors) (**Figure 5A**). Furthermore, we also assessed the correlation between *SIGLEC15* mRNA expression and 56 previously defined immune-related molecular features; the expressions of 11 molecular features were significantly higher in the high *SIGLEC15* group, including Dendritic Activated, IFN gamma Response, and Leukocyte Fraction (**Supplementary Figures S2A–K**). We further evaluated the correlation between *SIGLEC15* expression and immunocyte infiltration and observed that *SIGLEC15* significantly positively correlated with the infiltration of B cells, CD4 T cells, macrophages, neutrophils, and dendritic cells (**Figure 5B**). Moreover, the high *SIGLEC15* group employed a higher stromal score, immune score, and estimate score (**Figure 5C**). We also used the ssGSEA algorithm to calculate immunocyte infiltration; it was easy to see that all of the 16 immune cells and 13 immune-related pathway scores were significantly upregulated in the high *SIGLEC15* group (**Figures 5E, F**). Although the high *SIGLEC15* group had a higher proportion of immunocytes and an elevated level of immune checkpoints, we also observed that the high *SIGLEC15* group linked with an increased score of TIDE, immune exclusion by TIDE database (**Figure 5D**). Moreover, by integrating some known gene sets correlated with exhausted immunity, we found that although patients with high *SIGLEC15* had a high proportion of immunocytes, they also had higher scores of immune checkpoint blockade (ICB) resistance, exhausted CD8, T-cell exhaustion, immune checkpoint, and T-cell regulatory, which would lead to immune exhaustion. So, high *SIGLEC15* subgroup patients met immune exhaustion, which indicated that patients with a low *SIGLEC15* expression could benefit more from Immune checkpoint inhibitor (ICI) therapy than patients with a high *SIGLEC15* expression (**Supplementary Figure S2L**). We also figured out that mainly immune cells express *SIGLEC15*, especially on Monocytes and Macrophages (**Supplementary Figure S3A**).

### *SIGLEC15* mRNA expression correlates with methylation

**Figure 6:**
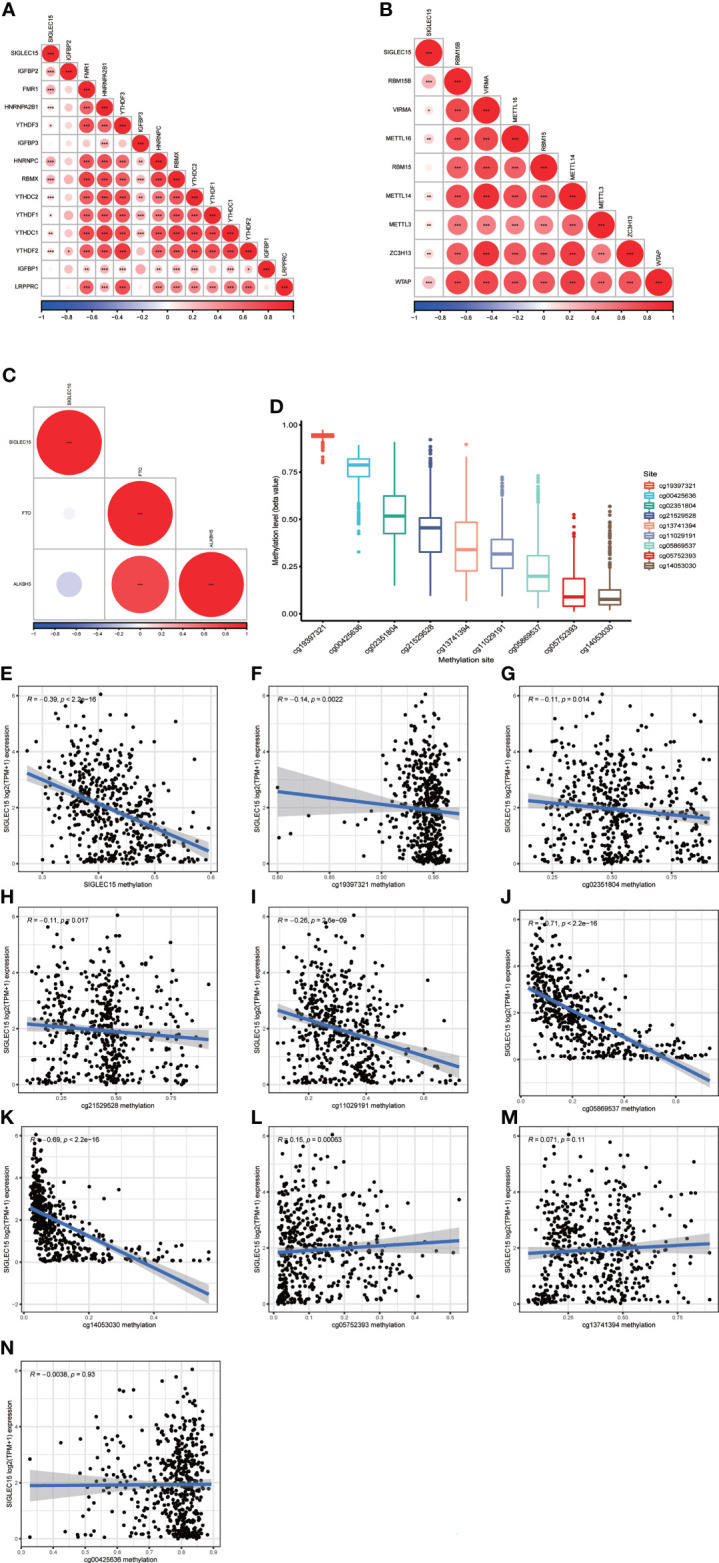
*SIGLEC15* mRNA expression correlation with DNA methylation and m6A regulator mRNA expression. **(A–C)** *SIGLEC15* mRNA expression correlation with **(A)** m6A readers, **(B)** writers, and **(C)** erasers. **(D)** Each CpG methylation site level of *SIGLEC15*. **(E)** *SIGLEC15* mRNA expression correlation with averaged CpG methylation site. **(F–N)** *SIGLEC15* mRNA expression correlation with DNA methylation of each CpG methylation site.

m6A RNA methylation was a kind of epigenetic modification measured by methyltransferases, demethylases, and binding proteins, which were also called “writers,” “erasers,” and “readers.” We conducted the relationship analysis of these regulated genes with *SIGLEC15* mRNA expression levels; it could be easily seen that the majority of reader genes had a significant positive correlation with *SIGLEC15* (**Figure 6A**), and all of the writer genes except RBM15 had a significant positive correlation with *SIGLEC15* (**Figure 6B**). However, eraser genes had no significant correlation with *SIGLEC15* (**Figure 6C**). We also analyzed the extent to which *SIGLEC15* mRNA expression correlated with CpG methylation and the whole CpG methylation site levels of *SIGLEC15*, which varied from a broad scope (**Figure 6D**). Interestingly, the averaged *SIGLEC15* promoter and body hypermethylation were associated with decreased *SIGLEC15* mRNA expression, indicated by a strong negative correlation coefficient (**Figure 6E**). Moreover, we measured each CpG methylation site level with *SIGLEC15* mRNA expression levels; we found that six out of nine sites had a negative correlation with *SIGLEC15*, and CpG methylation site cg05752393 had a positive correlation with *SIGLEC15* (**Figures 6F–L**). However, the CpG methylation site cg13741394 and cg00425636 had no significant correlation with *SIGLEC15* (**Figures 6M, N**).

### Clinical significance of *SIGLEC15*

**Figure 7:**
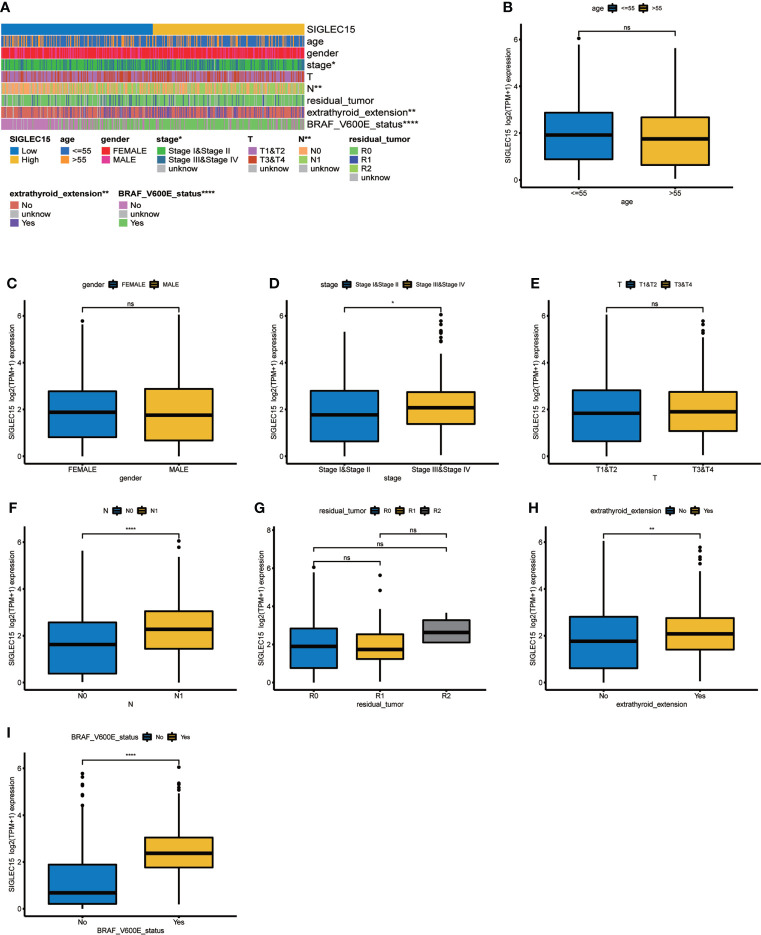
Clinical significance of *SIGLEC15*. **(A)** Heatmap of clinical feature correlation with *SIGLEC15*. **(B–I)** High and low *SIGLEC15* mRNA expression group difference in **(B)** Age, **(C)** Gender, **(D)** Stage, **(E)** T stage, **(F)** N stage, **(G)** Tumor residual size, **(H)** Extrathyroid extension, and **(I)** *BRAF* V600E status. ns, *, **, ***, and **** stand for p-value >0.05, p-value <=0.05, p-value <=0.01, pvalue <=0.001 and pvalue <=0.0001, respectively.

We quantified vital clinical feature associations with *SIGLEC15* mRNA expression in THCA, and stage, lymph node metastasis (N stage), extrathyroid extension, and *BRAF* V600E status were found to be positively correlated with *SIGLEC15* mRNA expression levels; other clinical factors (e.g., age, gender, residual tumor) indicated no significant relationship with *SIGLEC15* mRNA expression (**Figures 7A–I**). Furthermore, we analyzed the clinical feature associations with *SIGLEC15* IHC level, and we revealed that N stage and extrathyroid extension were positively related to *SIGLEC15* in **Table 2**. However, there was no significant relationship between *SIGLEC15* expression and overall survival (**Supplementary Figure S4A**).

**Table 2:** Relationship between clinicopathological characteristics and expression of *SIGLEC15*.

Parameter	N	H-Score ( $\bar{X}\pm \text{S}$ )	t-value	p*-*value
Gender				
Men	29	92.63 ± 30.19	0.525	0.601
Women	81	95.95 ± 29.02		
Age, years				
≤55	84	95.62 ± 27.23	0.355	0.723
>55	26	93.28 ± 35.49		
Extrathyroid extension				
No	67	88.13 ± 31.59	3.184	0.002
yes	43	105.72 ± 21.94		
Tumor focality				
Unifocal	76	94.30 ± 30.0	0.134	0.894
Multifocal	34	93.45 ± 31.98		
Lesion side				
Ipsilateral	77	92.81 ± 27.27	1.24	0.21
Bilateral	33	100.34 ± 33.21		
Lymph node metastasis				
N0	44	87.91 ± 28.68	2.31	0.035
N1a+N1b	66	99.84 ± 28.82		

H-Score, histochemistry score; N0, no lymph node metastasis; N1a, central lymph node metastasis; N1b, lateral cervical lymph node metastasis.

### Mutational analyses of *SIGLEC15* in thyroid carcinoma

**Figure 8:**
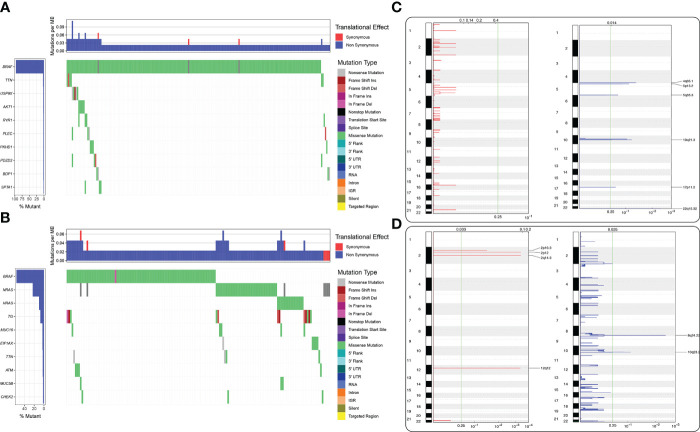
Differential mutational landscape in the high and low SIGLEC15 mRNA expression groups. **(A)** Genes were ranked according to the mutational frequency. SNV and Indel mutations in the high SIGLEC15 mRNA expression group. **(B)** Genes were ranked according to the mutational frequency. SNV and Indel mutations in the low SIGLEC15 mRNA expression group. **(C, D)** CNV landscape of **(C)** amplification and **(D)** deletion in the high and low SIGLEC15 mRNA expression groups; the chromosome was oriented vertically from top to bottom and GenePattern GISTIC2.0 q-value at each locus was placed from left to right. The green line displayed the cutoff value of q-value = 0.25.

No mutations were found in the MAF file of the gene *SIGLEC15* of THCA patients produced by varscan2 software obtained from The Cancer Genome Atlas, and GEO is Gene Expression Omnibus. We also investigated mutational profiles of low and high *SIGLEC15* groups; it could be clearly seen that more patient samples in the high *SIGLEC15* group had gene *BRAF* mutations. Moreover, the majority of mutations in patient samples of the high *SIGLEC15* group were located in gene *BRAF* (**Figure 8A**); patient samples in the low *SIGLEC15* group had mutations in genes *BRAF*, *NRAS*, and *HRAS* (**Figure 8B**), not focused as that in the high *SIGLEC15* group. The GISTIC2.0 results indicated that amplification displayed a lower frequency in the high *SIGLEC15* mRNA expression group compared to the low *SIGLEC15* expression group (**Figures 8C, D**) and similar frequency of deletion in the two groups (**Figures 8C, D**). Furthermore, we calculated the G-score, which also showed more amplification events in the low *SIGLEC15* expression group (**Supplementary Figures S5A, S5B**).

### DDR deficiency association with *SIGLEC15*

**Figure 9:**
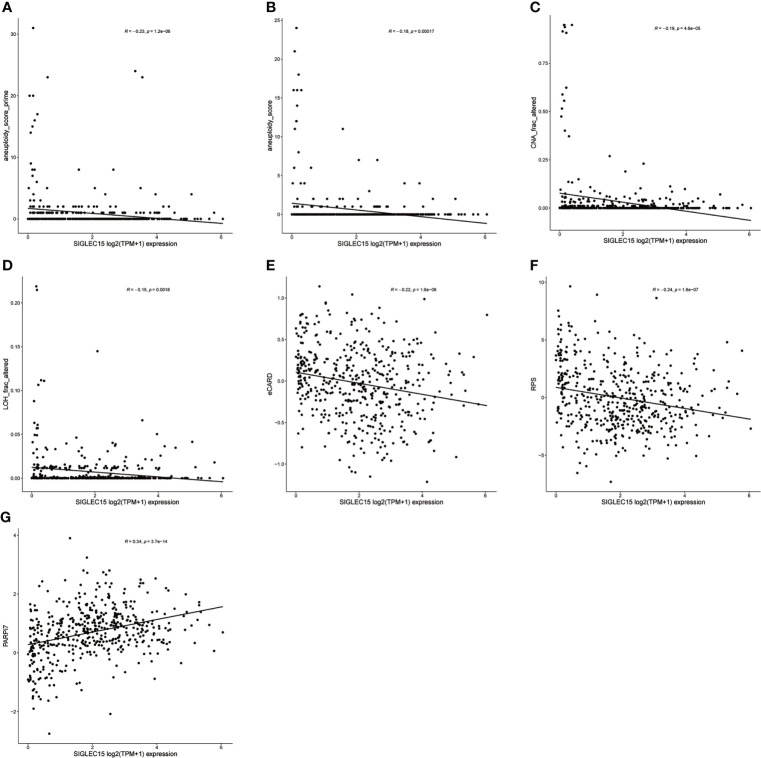
DDR deficiency score correlation with *SIGLEC15* mRNA expression. **(A–G)** *SIGLEC15* mRNA expression with score of **(A)** aneuploidy score prime, **(B)** aneuploidy score, **(C)** CNA frac altered, **(D)** LOH frac altered, **(E)** eCARD, **(F)** RPS, and **(G)** PARPi7.

DDR genes played vital roles in maintaining genomic stability, so the relationship between DDR deficiency scores and *SIGLEC15* was evaluated. We observed that many scores had a negative correlation with *SIGLEC15*, aneuploidy score prime (correlation coefficient = -0.23) (**Figure 9A**), aneuploidy score (correlation coefficient = -0.18) (**Figure 9B**), CNA frac altered (correlation coefficient = -0.19) (**Figure 9C**), LOH frac altered (correlation coefficient = -0.15) (**Figure 9D**), expression CDF trAnsform of Rank Distribution (eCARD) (correlation coefficient = -0.22) (**Figure 9E**), and repair proficiency scoring (RPS) (correlation coefficient = -0.24) (**Figure 9F**); nevertheless, PARPi7 (7-gene DNA repair deficiency expression signature) had a positive correlation with *SIGLEC15* mRNA expression (correlation coefficient = 0.34) (**Figure 9G**).

### Potential chemotherapy drugs for *SIGLEC15* determined thyroid carcinoma progress

**Figure 10:**
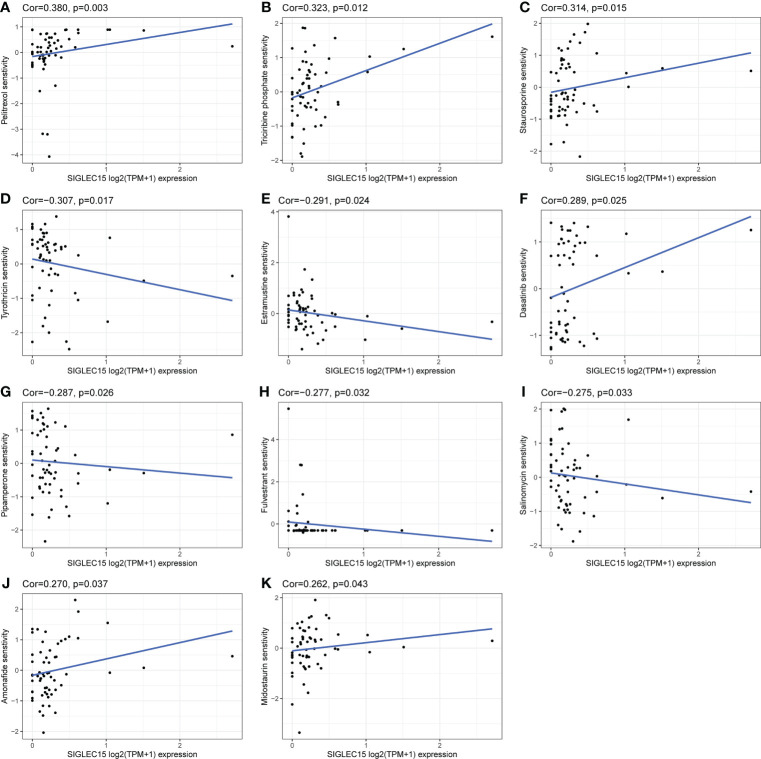
Potential effective chemotherapy drugs with *SIGLEC15*. **(A–K)** Correlation of *SIGLEC15* mRNA expression level and IC50 of different drugs based on the CellMiner database.

Combining chemotherapy drugs with a single-agent immune checkpoint therapeutic approach may enhance antitumor immune response and overcome primary resistance. We revealed that *SIGLEC15* mRNA expression was negatively associated with the IC50 of tyrothricin, estramustine, pipamperone, fulvestrant, and salinomycin and implied that these selected chemotherapeutic drugs may be suitable for the treatment of those with a high expression level of *SIGLEC15*, while selected chemotherapeutic drugs like pelitrexol, triciribine phosphate, staurosporine, dasatinib, amonafide, and midostaurin might exert an opposite effect for the treatment of those with a high expression level of *SIGLEC15* (**Figures 10A–K**).

## Discussion

Thyroid cancer was one of the most prevalent endocrine cancers with an elevated incidence rate over the past decades, and it was the fifth leading incidence of cancer in women (4). Although the low mortality and moderate prognosis were frequently mentioned, the recurrence and the complications were still obscure. In these years, immunotherapy was applied to the treatment of advanced differentiated thyroid cancer and anaplastic thyroid cancer, with some success (20). Despite the immense success of multiple antibody-based immune therapies targeting *PD-1*/*PD-L1* in common clinical regimens, there were still many non-responding patients (59). Since *PD-1*/*PD-L1* represented only one of many inhibitory immune checkpoints, exploration of other potential new immune modulators that could be blocked to expand the success of cancer immunotherapy and promote the anticancer immune response is urgently needed.

In this study, the features of *SIGLEC15* in multi-omics data in THCA cases were comprehensively characterized for the first time. We revealed that *SIGLEC15* was overexpressed in THCA. Consistent with our result, previous studies through integrative data mining of *SIGLEC15* mRNA expression in human tumors showed that higher *SIGLEC15* levels were observed in colon adenocarcinoma and thyroid carcinoma (60), colon adenocarcinoma, esophageal carcinoma and thyroid carcinoma (61). Chen et al. found that *SIGLEC15*-knockout mice exhibited retarded tumor growth and prolonged survival compared to wild-type mice. Hao et al. (62) showed that *SIGLEC15* mRNA expression was not associated with the prognosis of early non-small cell lung cancer. Liang et al. (63) proved that high *SIGLEC15* mRNA expression was not related to either overall survival or disease-free survival in patients with non-small cell lung cancer. Quirino et al. (64) found that *SIGLEC15* was also not correlated with either overall survival or relapse-free survival in gastric adenocarcinoma. In contrast, *SIGLEC15* positivity had better disease-specific survival and progression-free survival compared to *SIGLEC15* negativity in pancreatic ductal adenocarcinoma (30). Nevertheless, Li et al. (65) demonstrated that patients with a high *SIGLEC15* mRNA expression had worse overall survival and disease-free survival than patients with low *SIGLEC15* in the PACA-AU database, but no association was observed between *SIGLEC15* and prognosis in their own microarray cohort. Thus, it remained to be determined which biomarkers (*SIGLEC15* IHC or mRNA) could better guide patient selection for treatment response to *SIGLEC15*-associated therapy, and there has exited a companion diagnostic assay of SIGLEC5 by immunohistochemical was conducted by Shafi et al. (66). In addition, the pan-cancer analysis and our result showed that the expression of *SIGLEC15* may play distinctive roles in different human cancers, such as acting as an immunosuppressor in “hot tumor” non-small cell lung cancer, so anti-*SIGLEC15* therapy was suitable for such tumor. Meanwhile, in our study, we proved that increased *SIGLEC15* expression positively correlated with more extrathyroid extension and lymph node metastasis, indicating the vital role of *SIGLEC15* in the malignant progression of THCA; thus, THCA patients may benefit from the block antibodies for *SIGLEC15*.

Hu et al. (31) indicated that anti-*SIGLEC15* therapy could be feasible for bladder cancer treatment as *SIGLEC15* related to a non-inflamed TME in bladder cancer. Chen et al. (30) revealed that *SIGLEC15* was related to a low density of Regulatory T cells (Tregs) and CD45RO T cells, and Wang et al. (27) also showed that *SIGLEC15* suppressed antigen-specific T-cell responses. Liu et al. (67) demonstrated that *SIGLEC15* promoted the migration of hepatoma cells through regulating the *CD44* protein stability in liver cancer. Li et al. showed that *SIGLEC15* performed immunosuppressive functions by directly inducing immunosuppressive tumor-associated macrophages (TAMs) *via* binding to α-2,3 sialic acid. Liu et al. (32) clarified the importance of LINC00973-*miR-7109*- *SIGLEC15* regulation axis in immune evasion of clear-cell renal cell carcinoma. Our results showed that immunomodulators such as HLA class I and II and chemokines were upregulated in the higher *SIGLEC15* group, which were vital molecules that induced adaptive immune responses (68); our results also showed a significant positive correlation between *SIGLEC15* and *CD44*. In the cancer immunity cycle process, there existed seemingly contradictory results, step 1 (release of cancer cell antigens), step 6 (recognition of cancer cells by T cells), and step 4 (Th17 cell recruiting) were downregulated in the higher *SIGLEC15* group; nevertheless, the higher *SIGLEC15* group also met immune exhaustion and thus may induce an immune escape environment for patients and finally responded less to ICB therapy. In addition, patients who were in the higher *SIGLEC15* group had more *BRAF* V600E mutation, which was a poor prognosis factor in THCA. Previous studies evidenced that treatment with inhibitors that target the *BRAF* kinase combined with anti-*PD-1* therapy improved antitumor immunity in *BRAF*-mutant melanoma (69, 70). Clinical trial NCT02130466 showed that combined dabrafenib (a *BRAF* inhibitor) plus trametinib (a *MEK1* and *MEK2 (MEK1/2)* inhibitor) plus pembrolizumab (an anti-*PD-1* antibody) had more antitumor activity than dabrafenib plus trametinib plus placebo (71, 72). Our result also found some potent chemotherapy drugs for the high and low *SIGLEC15* groups, so this may provide a rationale for using immuno-oncology agent combinations for THCA patients. The mentioned above result also signified the complex TME in THCA.

DNA methylation and m6A methylation were two epigenetic mechanisms for the regulation of gene expression in eukaryotes and acted as vital regulators in cancer (73–75). We firstly fully described the negative correlation of DNA methylation and expression of *SIGLEC15* and prognosis in THCA in detail. Another pan-cancer also revealed the negative correlation in bladder cancer, uterine corpus endometrial carcinoma, breast invasive carcinoma, pancreatic ductal adenocarcinoma, etc. (61). We also firstly revealed the m6A methylation regulator relationship with *SIGLEC15*; regulating the expression of *SIGLEC15 via* methylation in cancer may be another road.

In addition to its function in immune regulation, Chen et al. (30) demonstrated that *SIGLEC15* mRNA expression had a positive correlation with high *BRCA1* status by IHC, and combining *SIGLEC15* with different DDR molecular statuses may be a potential prognosis predictor. Read et al. (76) revealed that elevated pituitary tumor transform gene (PTTG) and pituitary tumor transforming gene binding factor (PBF) modulate DNA damage response genes in thyroid cancer. We found that *SIGLEC15* was negatively related to *BRCA1* in the mRNA level but no correlation with *BRCA2*. We also found that high *SIGLEC15* had a negative correlation with DDR deficiency scores, such as aneuploidy score, CNA frac altered, and LOH frac altered, and these results implied that *SIGLEC15* may affect thyroid cancer progression through interacting with DDR genes.

There were some limitations in the current work. Firstly, more experiments need to be done to figure out the cellular mechanism of *SIGLEC15* in THCA. Secondly, there was no animal model experiment, so mouse models and either humanized or spontaneous but containing genomic features relevant to THCA animal models were needed to prove the results. Therefore, animal models, clinical verification data from multiple centers, and prospective studies were required to confirm our findings.

## Conclusion

In conclusion, we found that *SIGLEC15* mRNA expression was upregulated in tumor tissue and validated that by TMA. Clinical feature integration supported that increased *SIGLEC15* mRNA expression promoted extrathyroid extension and lymph node metastasis, and elevated *SIGLEC15* group patients met immune exhaustion. Specifying the role of *SIGLEC15* in THCA could represent a potential next-generation cancer immunotherapy option for patients.

## Data availability statement

The original contributions presented in the study are included in the article/**Supplementary Material**. Further inquiries can be directed to the corresponding authors.

## Ethics statement

The studies involving human participants were reviewed and approved by Ethics Committee of Zhejiang Cancer Hospital. The patients/participants provided their written informed consent to participate in this study.

## Author contributions

XFH, CC, XBL, and XDH have contributed equally to this work. XDH, XBL conceptualized and designed this study. CC, XFH Provision and collection of study materials, XFH, XBL, and XDH wrote the first draft of the manuscript. All authors contributed to the article and approved the submitted version.

## Funding

This study was funded by the Medical and Health Research Program of Zhejiang Province 2022 Grant/Award Number: 2022PY005.

## Conflict of interest

The authors declare that the research was conducted in the absence of any commercial or financial relationships that could be construed as a potential conflict of interest.

## Publisher’s note

All claims expressed in this article are solely those of the authors and do not necessarily represent those of their affiliated organizations, or those of the publisher, the editors and the reviewers. Any product that may be evaluated in this article, or claim that may be made by its manufacturer, is not guaranteed or endorsed by the publisher.

## Supplementary material

Supplementary Figure 1 Analysis of expression of *SIGLEC15*. **(A)** pan-cancer mRNA expression of *SIGLEC15* between tumor and normal tissues from PCAWG database. **(B)** mRNA expression of *SIGLEC15* between paired tumor and normal tissues from THCA (Thyroid carcinoma). **(C)** Correlation between mRNA expression of *SIGLEC15* and *CD44*.

Supplementary Figure 2
**(A-K)** Differential enrichment scores of immune signatures between high and low *SIGLEC15* groups. **(L)** Enrichment scores of immune exhausted scores in low and high *SIGLEC15* groups.

Supplementary Figure 3 Single-cell analysis exploration of the expression cell type of *SIGLEC15*.

Supplementary Figure 4 Kaplan-Meier survival curves between *SIGLEC15* expression and survival.

Supplementary Figure 5
**(A, B)** Comparison of amplification and deletion of CNV in high and low *SIGLEC15* mRNA expression groups.

Supplementary Table 1 Correlation details in expression of *SIGLEC15* and *CD44*.

## Abbreviations

Abbreviations: ACC, Adrenocortical carcinoma; BLCA, Bladder Urothelial Carcinoma; BRCA, Breast invasive carcinoma; CESC, Cervical squamous cell carcinoma and endocervical adenocarcinoma; CHOL, Cholangiocarcinoma; COAD, Colon adenocarcinoma; COADREAD, Colon adenocarcinoma/Rectum adenocarcinoma Esophageal carcinoma; ESCA, Esophageal carcinoma; GBM, Glioblastoma multiforme; GBMLGG, Glioma; HNSC, Head and Neck squamous cell carcinoma; KICH, Kidney Chromophobe; KIPAN, Pan-kidney cohort (KICH+KIRC+KIRP); KIRC, Kidney renal clear cell carcinoma; KIRP, Kidney renal papillary cell carcinoma; LAML, Acute Myeloid Leukemia; LGG, Brain Lower Grade Glioma; LIHC, Liver hepatocellular carcinoma; LUAD, Lung adenocarcinoma; LUSC, Lung squamous cell carcinoma; OV, Ovarian serous cystadenocarcinoma; PAAD, Pancreatic adenocarcinoma; PCPG, Pheochromocytoma and Paraganglioma; PRAD, Prostate adenocarcinoma; READ, Rectum adenocarcinoma; STAD, Stomach adenocarcinoma; SKCM, Skin Cutaneous Melanoma; STES, Stomach and Esophageal carcinoma; TGCT, Testicular Germ Cell Tumors; THCA, Thyroid carcinoma; UCEC, Uterine Corpus Endometrial Carcinoma; UCS, Uterine Carcinosarcoma; ALL, Acute Lymphoblastic Leukemia; WT, High-Risk Wilms Tumor.
