# Supplementary figures and images for: Unveiling the molecular features, relevant immune and clinical characteristics of SIGLEC15 in thyroid cancer
Source: Front Immunol. 2022 Sep 9;13:975787. doi: 10.3389/fimmu.2022.975787 (PMC9500188; doi:10.3389/fimmu.2022.975787)

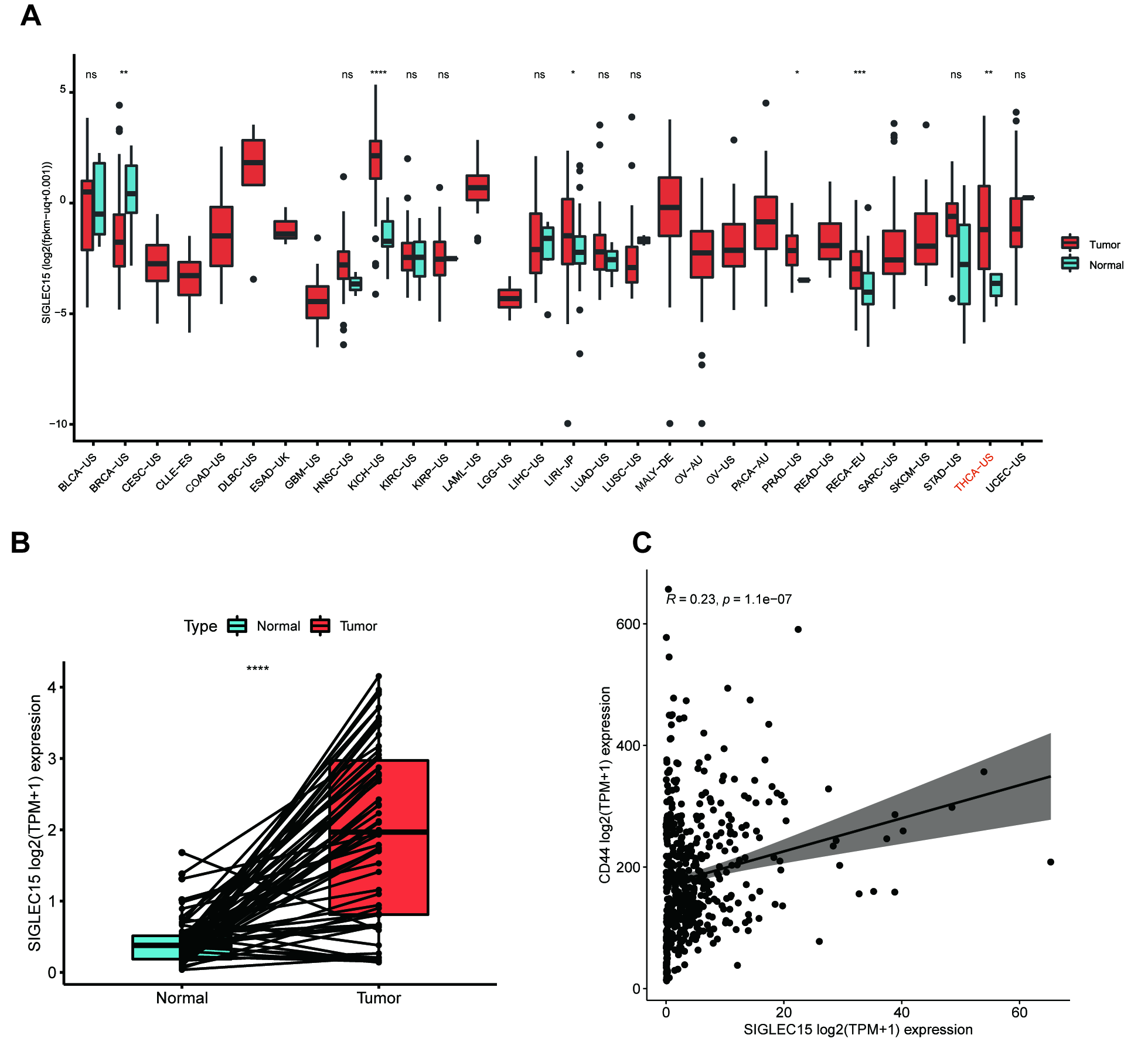

Supplement: Supplementary Figure 1 — Analysis of expression of SIGLEC15. (A) pan-cancer mRNA expression of SIGLEC15 between tumor and normal tissues from PCAWG database. (B) mRNA expression of SIGLEC15 between paired tumor and normal tissues from THCA (Thyroid carcinoma). (C) Correlation between mRNA expression of SIGLEC15 and CD44. [file Image_1.tif]

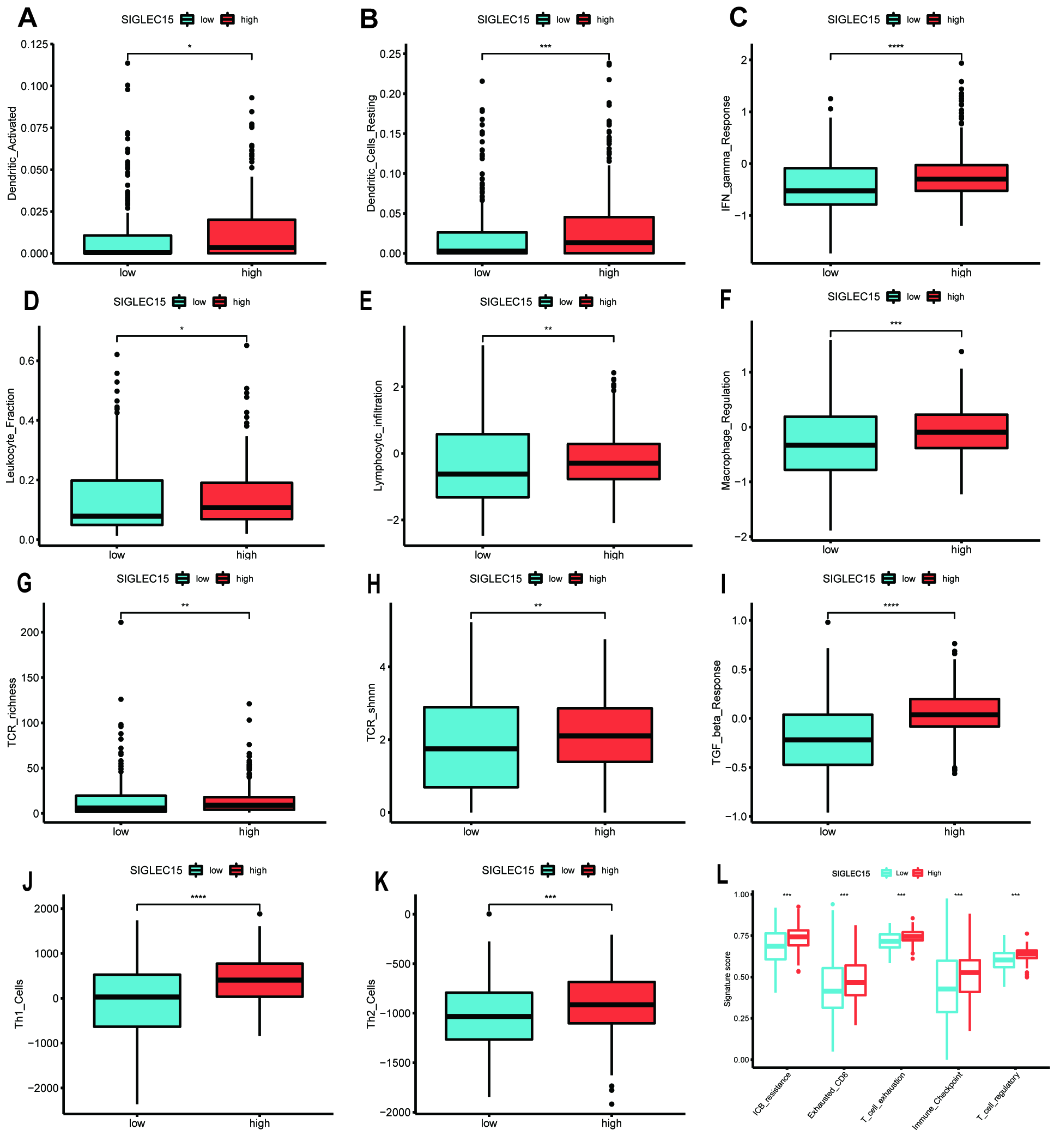

Supplement: Supplementary Figure 2 — (A-K) Differential enrichment scores of immune signatures between high and low SIGLEC15 groups. (L) Enrichment scores of immune exhausted scores in low and high SIGLEC15 groups. [file Image_2.tif]

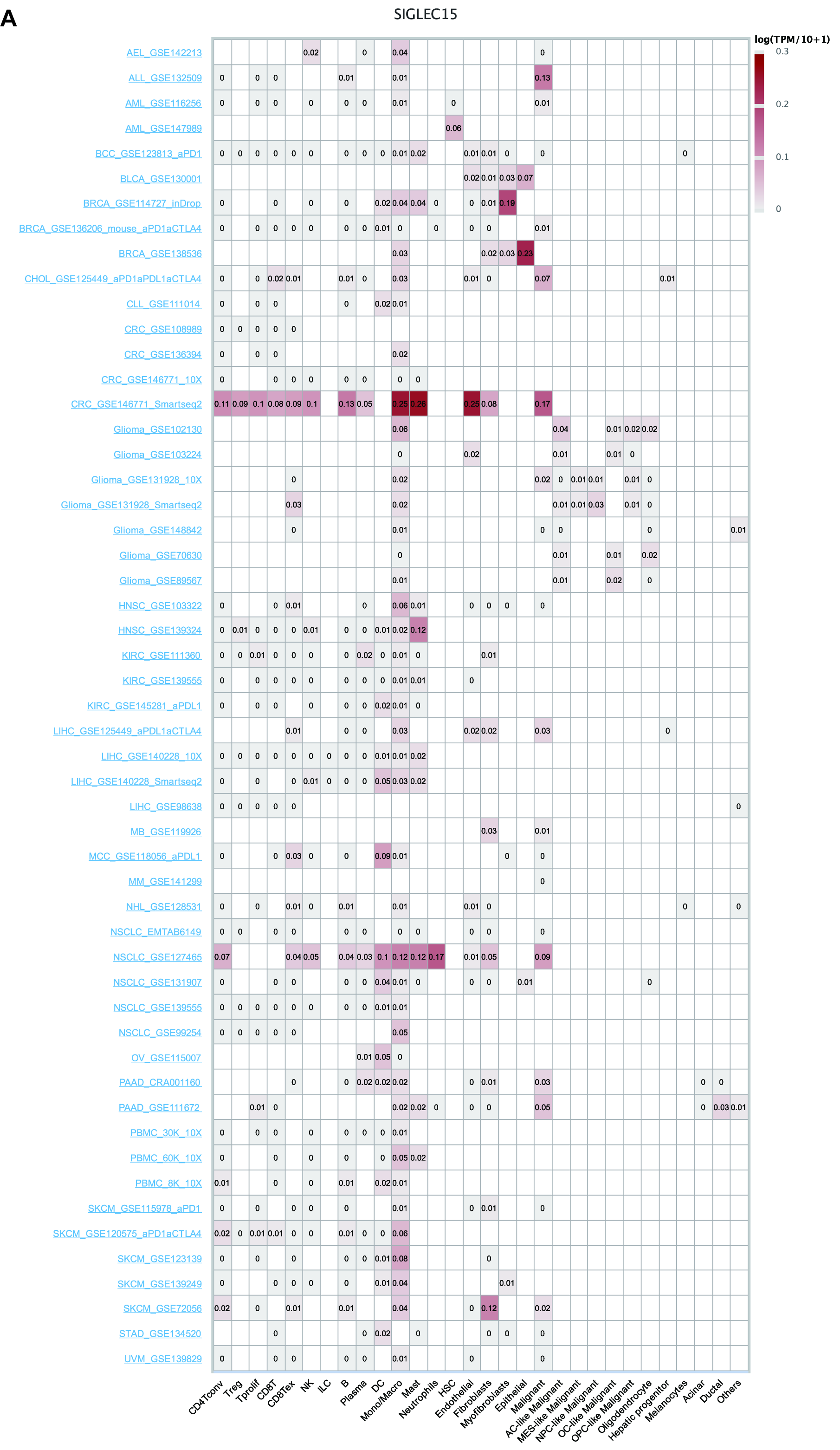

Supplement: Supplementary Figure 3 — Single-cell analysis exploration of the expression cell type of SIGLEC15. [file Image_3.tif]

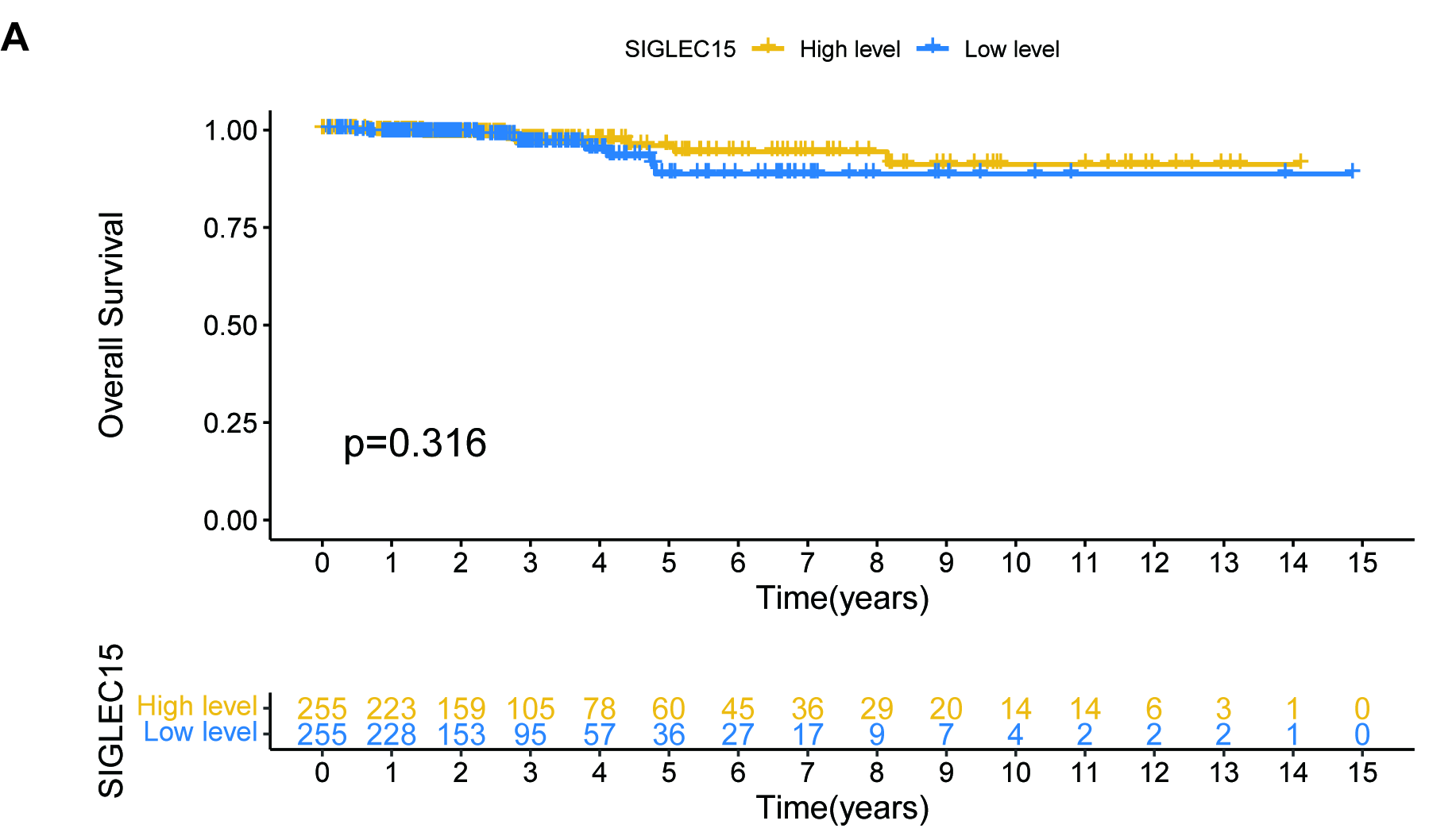

Supplement: Supplementary Figure 4 — Kaplan-Meier survival curves between SIGLEC15 expression and survival. [file Image_4.tif]

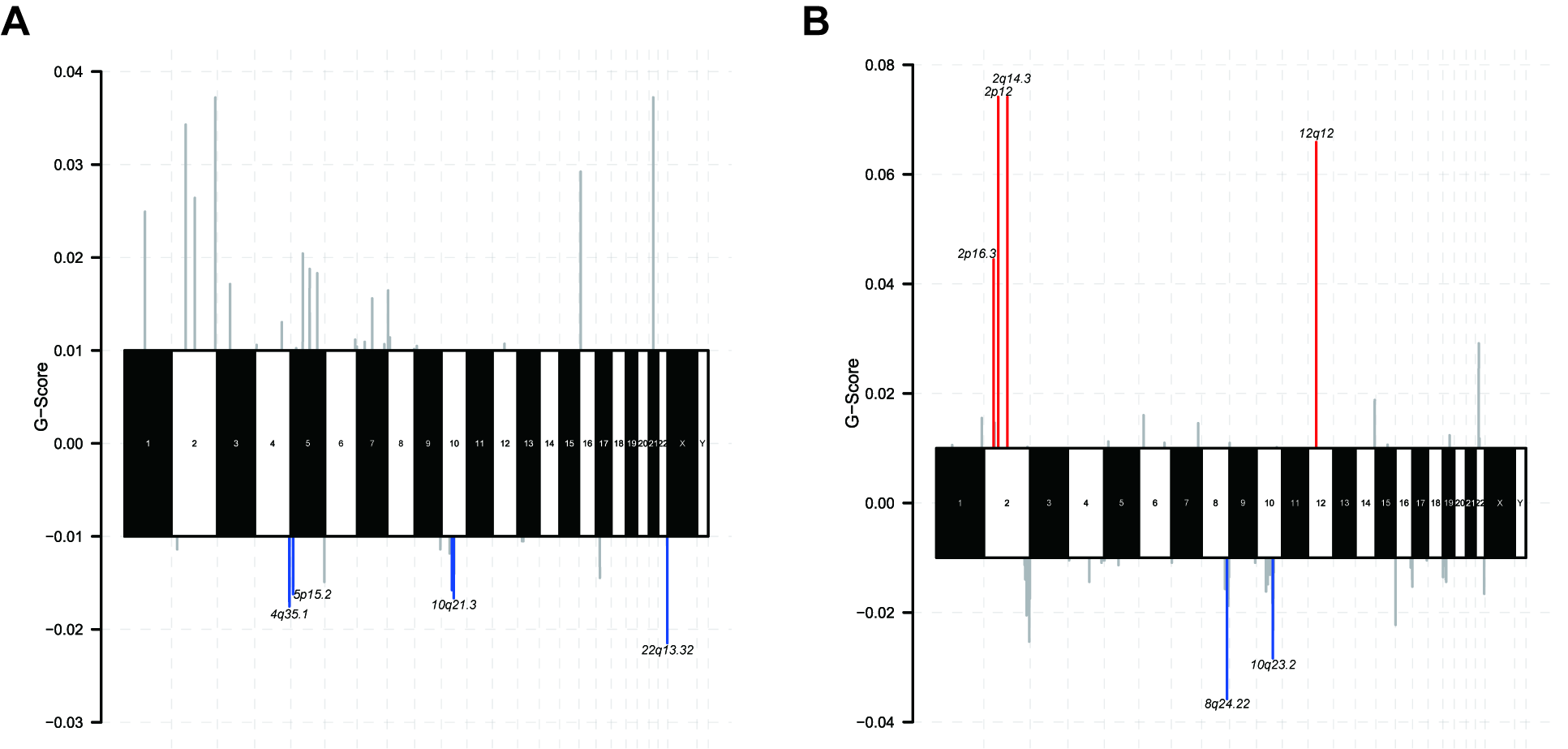

Supplement: Supplementary Figure 5 — (A, B) Comparison of amplification and deletion of CNV in high and low SIGLEC15 mRNA expression groups. [file Image_5.tif]
